# Assessment of Methods to Estimate Resting and Total Energy Expenditure in Children and Adolescents with Cerebral Palsy: A Scoping Review

**DOI:** 10.3390/healthcare14193295

**Published:** 2026-10-02

**Authors:** Jorge Abraham García-Íñiguez, Andrea Anaís García-Contreras

**Affiliations:** 1 Instituto de Nutrición Humana, Centro Universitario de Ciencias de la Salud, Universidad de Guadalajara, Guadalajara 44340, Jalisco, Mexico; j.garcia@edu.uag.mx; 2 Escuela de Nutrición, Universidad Autónoma de Guadalajara, Zapopan 45129, Jalisco, Mexico

**Keywords:** energy expenditure, resting energy expenditure, total energy expenditure, basal metabolic rate, doubly labeled water, cerebral palsy, children, indirect calorimetry

## Abstract

**Background/Objectives**: Accurate assessment of resting energy expenditure (REE) and total energy expenditure (TEE) in children with cerebral palsy (CP) remains challenging. This review aimed to map and synthesize the existing evidence on methods used to estimate REE and TEE in children and adolescents with CP and identify their limitations and research gaps. **Methods**: A structured search was conducted in PubMed, Scopus, SciELO, EBSCO, and Embase for English or Spanish language articles published with no restrictions on publication date until September 2026. Studies that included children or adolescents from 0 to 19 years with a confirmed diagnosis of CP and that reported measured or estimated REE, basal metabolic rate (BMR), or TEE were included. **Results**: Nineteen studies met the inclusion criteria. Indirect calorimetry (IC) was the most frequently used method for measuring REE (n = 11, 57.9%), whereas doubly labeled water (DLW) was the reference method for TEE (n = 6, 31.6%). Standard predictive equations developed for TDC were used in three studies (15.8%) and frequently overestimated REE. In contrast, CP-specific predictive equations were less used and showed better estimations against reference methods. BIA-derived estimates, anthropometric approaches, and biomarker-based models remain insufficiently validated. **Conclusions**: IC remains the reference method for individualized assessment of REE, whereas DLW is the reference method for measuring TEE. Predictive equations for TDC are not recommended for children with CP. Further validation studies comparing BIA-derived estimates and CP-specific equations against reference methods are needed, particularly across Gross Motor Function Classification System (GMFCS) levels, nutritional status, and feeding method.

## 1. Introduction

Cerebral palsy (CP) is one of the most common causes of motor disability in childhood and is defined as a group of disorders affecting movement and posture, attributed to non-progressive disturbances that occurred in the developing fetal or infant brain [1].

Children and adolescents with CP frequently present feeding difficulties, inadequate energy intake, reduced mobility, altered growth or inappropriate weight gain, and increased risk of malnutrition. These factors may contribute to altered resting energy expenditure (REE) [2,3]. REE represents the energy required to maintain essential physiological functions. Although closely related to basal metabolic rate (BMR), REE is measured under less restrictive conditions and is more commonly assessed in clinical practice [4].

Accurate assessment of energy expenditure (EE) in children with CP is one of the fundamental components that may contribute to the estimation of energy requirements.

Individualized assessment of EE in children and adolescents with CP is relevant due to the substantial heterogeneity in motor impairment, mobility, body composition, and nutritional status that characterizes this population, all of which may contribute to considerable interindividual variability in EE and may limit the accuracy of generalized approaches to estimating energy needs.

Objective assessment of EE, when interpreted alongside dietary intake, growth, body composition, and nutritional status, may provide valuable information to support personalized nutritional interventions promoting optimal growth and reducing the risk of both undernutrition and overnutrition, thereby potentially contributing to reduced morbidity and improved quality of life [4].

Estimating REE and total energy expenditure (TEE) in this population remains a clinical challenge, as energy requirements are influenced by multiple factors, including CP subtype, Gross Motor Function Classification System (GMFCS) level, muscle tone, nutritional status, altered body composition, and neurological comorbidities [5].

Several methods are available for assessing EE in the pediatric population. However, one of the main limitations in clinical practice is the absence of a clear and uniform consensus regarding the most appropriate approach for EE determination in children with CP [6].

Predictive equations originally developed for healthy populations, such as those proposed by Schofield, Harris–Benedict, or the World Health Organization (WHO) equation, continue to be widely used in clinical practice because of their simplicity and accessibility. However, their individual-level accuracy in children with CP is limited. These equations were not developed specifically for pediatric CP populations and may not adequately account for differences in body composition, growth, mobility, and motor impairment [6]. Consequently, they frequently tend to underestimate or overestimate energy requirements, particularly in children with severe functional limitations [7].

Some CP-specific equations have been developed to improve energy estimation in this population, including those proposed by Krick et al. [8] for energy requirements, Rieken et al. [9] for TEE, and Penagini et al. [7] and Borsani et al. [4] for REE. However, these equations have been less explored.

Indirect calorimetry (IC) has been recommended as the most accurate method to measure REE, although its use is limited by high cost and the need for specialized equipment and trained personnel [10]. Moreover, IC measurements may be affected by several factors such as involuntary movements, irregular breathing patterns, and difficulty maintaining a stable resting state, all of which are common in children with CP [4].

Previous studies have demonstrated variations in both REE and TEE among children with CP and healthy children [3,11,12,13,14,15,16,17,18,19]. Given the ongoing controversy, the limited number of comprehensive reviews, and the absence of consensus regarding the estimation of REE and TEE in children and adolescents with CP, as well as the clinical implications of inaccurate energy estimation, this scoping review aimed to map and synthesize the available evidence on predefined methods—IC, doubly labeled water (DLW), bioelectrical impedance analysis (BIA), and predictive equations—used to measure or estimate REE and TEE in children and adolescents with CP, as well as to identify methodological limitations and knowledge gaps. Based on the mapped evidence, an evidence-informed algorithm was proposed to support the assessment of EE in this population.

## 2. Materials and Methods

### 2.1. Search Strategy

This scoping review was conducted and reported in accordance with the Preferred Reporting Items for Systematic Review and Meta-Analysis Scoping Review (PRISMA-ScR) guidelines [20]. The protocol for this scoping review was registered in the Open Science Framework (OSF; registration number 8xh6s; https://osf.io/8xh6s/; accessed on 5 August 2026).

A literature search was performed to identify studies that measured or estimated REE and TEE in children with CP. The search was conducted in PubMed, Scopus, SciELO, EBSCO, and Embase databases. The search strategy included the following terms: (“cerebral palsy”) AND (“predictive equations” OR “indirect calorimetry” OR “bioelectrical impedance analysis” OR “bioimpedance” OR “doubly labeled water”) AND (“resting energy expenditure” OR “total energy expenditure” OR “basal metabolic rate” OR “metabolic rate”). The full search strategy is presented in Supplementary Table S1. No restrictions on publication date were applied during the literature search to capture a full range of available evidence. The final search update was performed in September 2026. Consistent with the exploratory purpose of this scoping review, no formal risk-of-bias assessment was conducted. Therefore, the findings should be interpreted as a mapping of the available evidence rather than as definitive comparative estimates of method accuracy.

### 2.2. Study Selection

**Figure 1:**
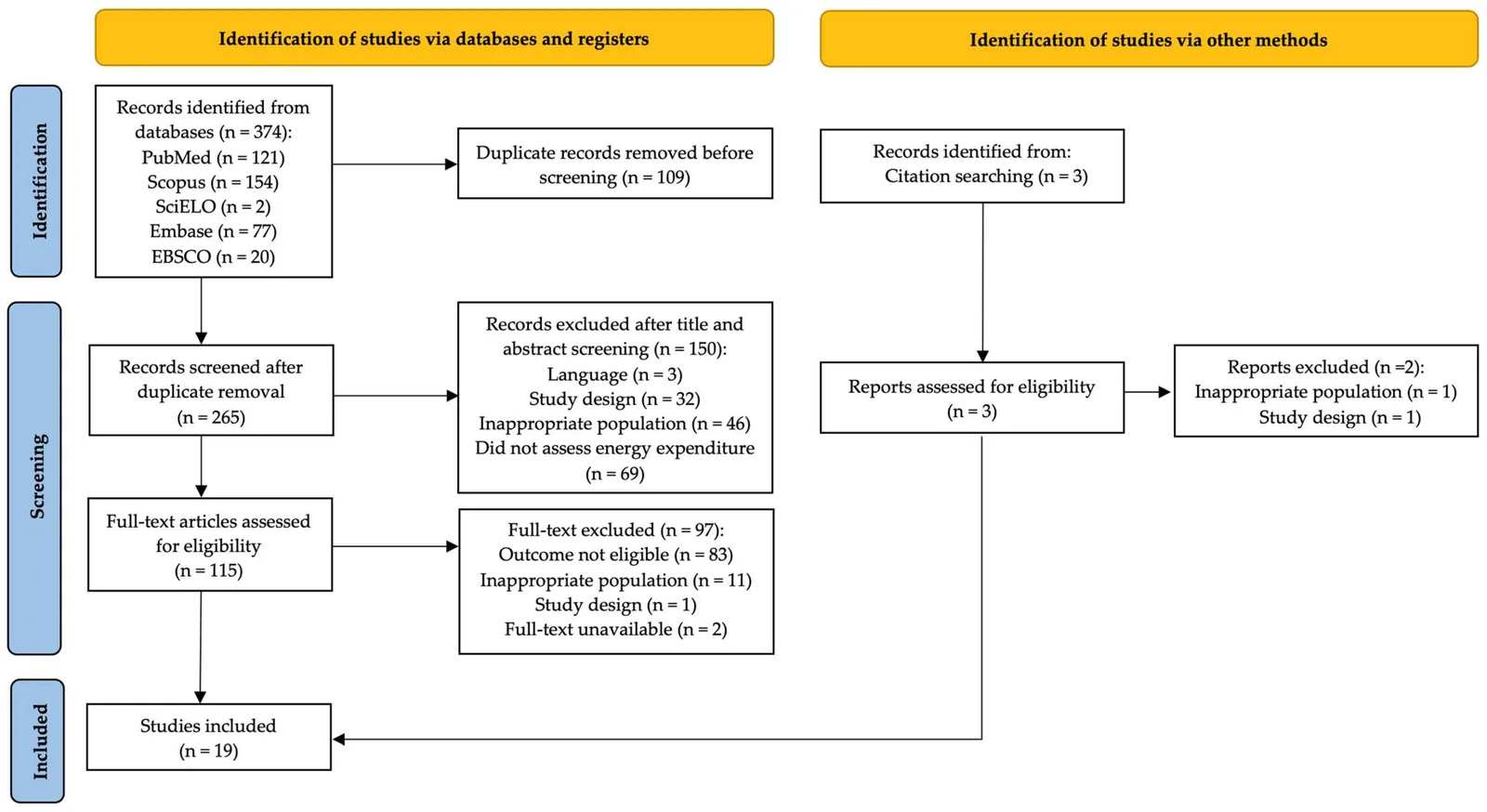
PRISMA flowchart of the study selection process.

The literature search was conducted by two researchers who independently screened titles and abstracts based on predefined eligibility criteria and subsequently assessed potentially eligible full-text articles. Disagreements between reviewers were resolved through discussion until consensus was reached. Duplicates were removed using Zotero (v. 7.0.32). A total of 374 records were identified through database searching; 109 of these were duplicates. After removing duplicates, 265 records were screened and assessed for relevance based on titles and abstracts. Of these, 150 records were excluded, and 115 full-text reports were assessed for eligibility. Ninety-seven records were excluded. As an additional citation-searching step, the studies specifically on children with CP included in the systematic review by Luo et al. [21] were cross-checked against the studies retrieved through our database searches. Three additional potentially eligible studies that had not been identified by the electronic search strategy were identified and assessed for eligibility. One study met the inclusion criteria and was included in the review. Finally, 19 studies were included (Figure 1).

### 2.3. Eligibility Criteria

Studies were eligible if they (1) included participants aged 0–19 years with a confirmed diagnosis of CP; (2) reported measured or estimated REE, BMR, or TEE; (3) clearly described the predefined methods (IC, DLW, BIA, and predictive equations); (4) were written in English or Spanish; and (5) used an observational, cross-sectional, longitudinal, or interventional design. Reviews, editorials, letters, case reports, conference abstracts, and studies without extractable REE, BMR, or TEE values or with a population outside the age range or without CP were excluded.

### 2.4. Data Charting

Relevant data were extracted independently by two researchers, including country, age, GMFCS level, sample size, method(s) used to assess EE, reported REE, BMR or TEE values expressed in kcal/day and kcal/kg/day, energy intake of children with CP, REE, BMR, and TEE of typically developing children (TDC) expressed in kcal/day. The results were organized according to the primary method used to estimate EE, allowing comparison of their clinical applicability, strengths, and limitations.

### 2.5. Evidence Synthesis

Given the heterogeneity of the included studies, the evidence was synthesized descriptively and described according to the method used to measure or estimate EE. Methodological strengths and limitations were identified from the methods and results reported in each study, considering comparisons between methods, agreement or differences in energy expenditure estimates, population characteristics, and clinical applicability. The clinical algorithm was derived narratively from these findings and was intended as an evidence-informed summary rather than a formally validated clinical guideline.

## 3. Results

Nineteen studies published between 1992 and 2023 met the inclusion criteria for this review. The evidence base consisted predominantly of cross-sectional studies, whereas prospective and interventional studies were uncommon.

Most studies included children with GMFCS levels I–V (n = 6, 31.6%); six studies (31.6%) included children with moderate to severe CP, particularly GMFCS levels IV–V; one study (5.3%) included children with GMFCS levels I–II; and in six studies, the GMFCS level was not reported. Spastic quadriplegic CP (SQCP) was the most frequently reported clinical CP subtype.

IC was used in 11 studies (57.9%) and was the most common method for measuring REE, reinforcing its status as the clinical standard for REE assessment. In six studies (31.6%), the DLW method was used. BIA for estimating REE or TEE was used in five studies (26.3%). Predictive equations for TDC were used in three studies (15.8%), demonstrating variable applicability. CP-specific predictive equations were used in two studies (10.5%). Finally, five studies (26.3%) explored alternative estimation strategies, including height-based and weight-based formulas as well as hormonal models. Categories were not mutually exclusive because several studies used more than one method. General characteristics and EE outcomes of the included studies are presented in Table 1.

The methodological characteristics of the included studies are summarized in Supplementary Table S2. The studies differed in the devices, comparators, and statistical approaches used to assess EE. Predictive equations or models, when reported, and the main methodological limitations identified across studies are also presented in this table.

### 3.1. Energy Expenditure Measured by IC

IC was the most frequently used method for measuring REE [4]. Several studies demonstrated that children with CP, particularly among those with severe motor impairment and intractable epilepsy, had lower REE values compared to TDC [3,12,13,15,16]. The studies included in this review showed that REE measured by IC in children with CP ranged from 574.5 kcal to 1075 kcal/day, particularly lower in those with severe motor impairment. The variability in the REE values measured by IC in the studies included ranged from 59.3 to 79.4% of those reported in healthy controls. Direct comparison across studies was limited by differences in age, body size, nutritional status, GMFCS level, and measurement protocols.

According to the feeding method, Arrowsmith et al. [3] reported higher measured REE in tube-fed children than orally fed children (*p* < 0.001). In addition, energy intake in the tube-fed group exceeded twice its measured REE. In contrast, other studies demonstrated that gastrostomy-fed children had significantly lower REE than their orally fed counterparts [2,9].

### 3.2. Energy Expenditure Measured by DLW

The studies included in this scoping review using the DLW method showed that TEE in children with CP was approximately 58.1% to 84.4% of that of TDC [13,16,18,19]. TEE values determined by DLW in the studies included ranged from 856.5 kcal to 1676 kcal/day.

Rieken et al. [9] demonstrated that TEE values measured by DLW were significantly lower in children classified as GMFCS level V than in those classified as level IV, with a mean difference of 516 kcal. Using the DLW method, energy requirements in ambulant children with CP were comparable to those observed in TDC [19]. Conversely, non-ambulant children with CP exhibited lower energy requirements, averaging 18% below those of ambulant children with CP and 31% below those of TDC.

The study performed by Walker et al. [19] showed that energy intake was similar to TEE estimated by DLW in children with moderate-severe CP, while in children with mild CP, energy intake values were 14.8% lower than TEE. However, the study performed by Stallings et al. [16] in children with SQCP showed that energy intake was 30.9% higher than TEE.

### 3.3. Energy Expenditure Estimated by Predictive Equations Developed for Healthy Children

Commonly used equations, originally developed for healthy pediatric populations, included the WHO, Harris–Benedict, Schofield, RDA, and Mifflin–St. Jeor equations. Errors in estimation reached approximately 21% to 33% compared to reference methods and showed a tendency toward overestimation of EE in children with CP [7].

Two studies [6,8] evaluated the RDA equation for estimating energy requirements in children with CP. The study performed by Lee et al. [6] reported that the RDA equation overestimated REE by 1127.6 kcal/day compared with values measured by IC. In contrast, the study performed by Krick et al. [8] showed that the RDA equation overestimated energy requirements by 11.2 kcal/kg/day compared to the CP-specific equation developed by the same authors.

Penagini et al. [7] demonstrated a difference between REE estimated by predictive equations for healthy populations and measured by IC with a tendency towards overestimation that varied from 37 ± 224 kcal/day to 79 ± 226 kcal/day using the Harris–Benedict equation and the Schofield equation, respectively.

Borsani et al. [4] found a proportional bias between the IC and all predictive equations for estimating REE in children with CP, including the Schofield (weight), Schofield (weight and height), Harris–Benedict, Oxford, and WHO equations. Among the evaluated equations, the Mifflin–St Jeor equation demonstrated comparatively better performance in their population. Nevertheless, none of the evaluated equations achieved sufficient individual-level accuracy for routine clinical use [4,6,7,8,12,18].

### 3.4. Energy Expenditure Estimated by Predictive Equations Developed for CP-Specific Populations

Only two studies evaluated CP-specific predictive equations for estimating energy requirements. The study conducted by Lee et al. [6] reported that the equation proposed by Krick et al. [8] substantially overestimated REE, yielding values approximately twice those measured by IC (ICC = 0.048). However, this estimate was closer to the IC measurement than the estimate obtained using the RDA equation. In the same study, the equation proposed by Culley et al. [23] yielded the closest agreement with REE measured by IC. Conversely, Krick et al. [8] estimated energy requirements expressed as kcal/kg/day and found that values derived from the RDA equation were higher than those estimated by Krick’s equation.

### 3.5. Energy Expenditure Estimated by BIA

BIA was mainly used to assess body composition, particularly fat-free mass (FFM) and total body water (TBW). Four studies used BIA for estimating REE ranging from 688.7 kcal to 929.7 kcal/day. In the studies included, BIA-derived REE estimates varied from 71.8% to 93.7% of those reported in healthy controls.

Two studies [15,22] estimated TEE by BIA, reporting that REE represented approximately 71.4% to 72.3% of the TEE. Tomoum et al. [17] identified that children with CP had lower REE (estimated by BIA) than healthy children (*p* < 0.001). Sung et al. [26] demonstrated a progressive decline in REE estimated by BIA across increasing GMFCS levels in children with CP. Children with severe motor dysfunction (GMFCS IV–V) exhibited a mean REE of approximately 200 kcal/day lower than those with mild-to-moderate impairment (GMFCS I–III). In addition, a moderate negative correlation was observed between REE and GMFCS level (r = −0.45), suggesting higher GMFCS levels were associated with lower estimated REE [26].

### 3.6. Energy Expenditure Estimated by Clinical Approaches

Other methods for estimating REE and TEE, including empirical approaches based on height (kcal/cm/day), body weight (kcal/kg/day), and hormonal markers such as ghrelin, offer practical alternatives when reference methods are unavailable.

Height-based recommendations of approximately 11–14 kcal/cm/day have been proposed for children with CP, although their validity may vary according to mobility, age, and nutritional status [22].

The kcal/kg/day approach may be useful in cases of undernutrition [15]. Seven studies reported REE in kcal/kg/day with values ranging from 34.9 to 90.6 kcal/kg/day. Borsani et al. [4] developed a predictive nomogram based on weight and triceps skinfold thickness (TSF) to estimate REE in children with CP. Zenitani et al. [27] demonstrated that plasma ghrelin levels had a moderate correlation with REE/kg (R^2^ = 0.62, *p* < 0.001); additionally, they developed an equation to estimate REE based on plasma ghrelin levels.

### 3.7. Algorithm to Assess REE or TEE

**Figure 2:**
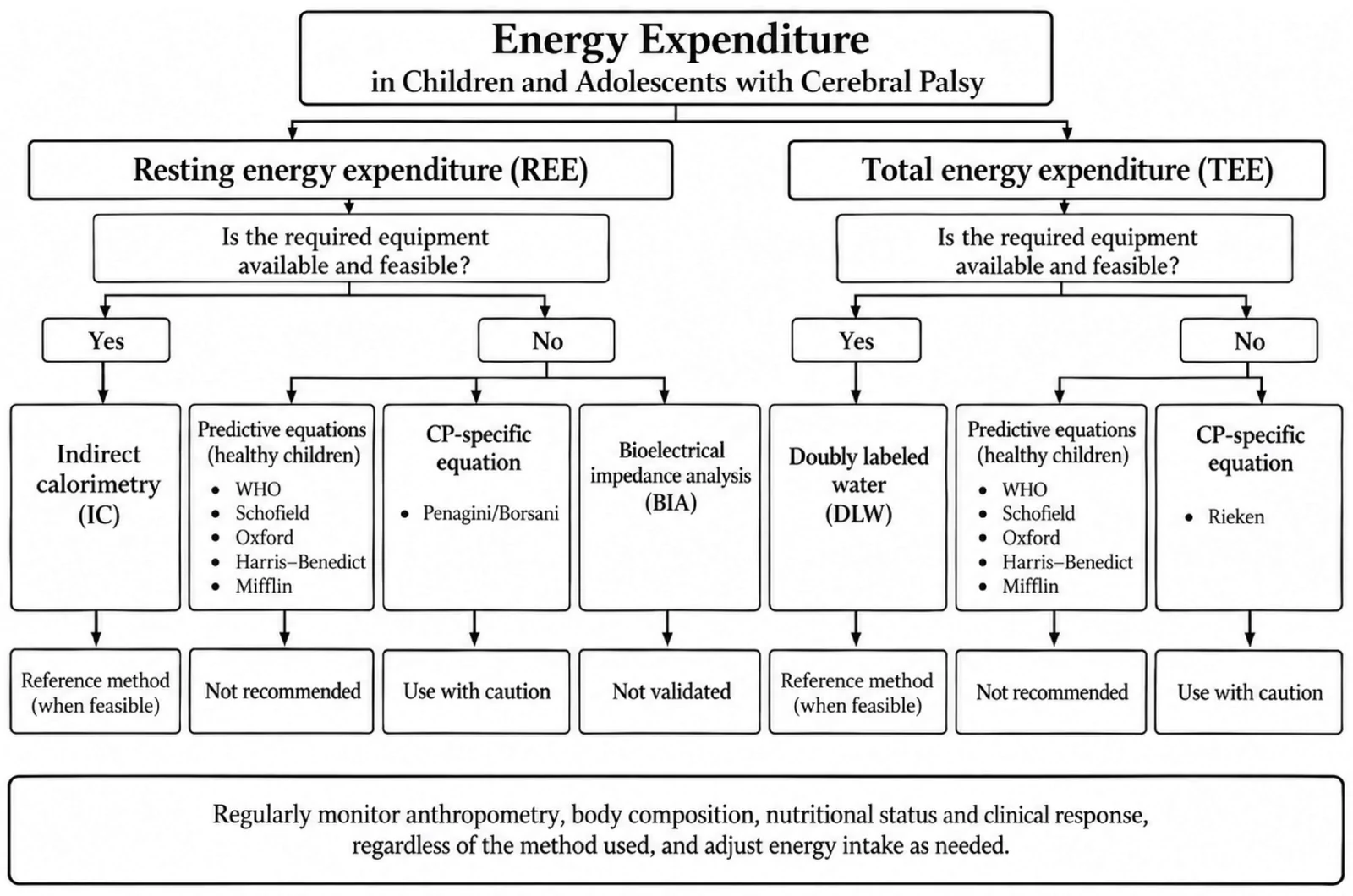
Proposed evidence-informed algorithm for selecting methods to assess REE or TEE in children with CP.

Figure 2 presents a practical algorithm based on the available evidence for selecting the most appropriate method for estimating EE in children with CP. IC should be considered the preferred method for measuring REE whenever the required equipment is available, whereas DLW remains the reference method for measuring TEE. When reference methods are unavailable, predictive equations for TDC are generally not recommended because of their poor accuracy in children with CP. CP-specific equations for estimating REE or TEE may be considered when no reference method is available. The present algorithm should be applied considering the individual characteristics of each patient (motor impairment, mobility, nutritional status, growth, body composition, physical activity, and clinical condition).

## 4. Discussion

This scoping review mapped and synthesized the evidence on predefined methods used to measure or estimate REE and TEE in children with CP as well as identified methodological limitations in the existing evidence.

The included studies revealed substantial heterogeneity in the REE and TEE in children with CP. This heterogeneity may be explained by differences in age, body size, GMFCS level, CP subtype, body composition, physical activity, and nutritional status.

It has been reported that REE in children with CP tends to be lower than that of TDC, mainly due to the decrease in muscle mass and low physical activity [28], and even more evident in children with severe CP [29]. Bell et al. [13] did not find a significant difference in REE between children with CP and TDC; this may be explained by the inclusion of ambulatory children classified as GMFCS levels I–II.

IC was the most frequently employed method for measuring REE because it directly quantifies gas exchange and can capture interindividual metabolic variability associated with spasticity, muscle atrophy, and reduced mobility [10,30,31,32].

Consistent evidence indicates that EE estimated by predictive equations developed for TDC shows significant bias when applied to children with CP, frequently leading to overestimation of energy requirements [7,12,31,32,33]. Several studies demonstrated low accuracy of WHO, Schofield, Harris–Benedict, and Oxford equations, particularly in patients with GMFCS levels IV–V [3,4,6,7]. Borsani et al. [4] demonstrated that the Mifflin–St Jeor equation showed comparatively better performance in a group of children with CP with GMFCS level V. This result could be due to the high prevalence of overweight/obesity in their sample, and the authors do not recommend its use in clinical practice. Bergqvist et al. [14] estimated REE using IC in 10 children with CP and found that the Schofield equation predicted 98% of the REE. Moreover, the sample in this study consisted of 40% ambulatory children. On the other hand, Penagini et al. [7] reported overestimations ranging from 37 to 79 kcal/day with correct classification rates ≤ 33% using the same equation, while Borsani et al. [4] documented estimation errors of approximately 22%. It has been demonstrated that the RDA equation overestimated the energy needs estimated in kcal/kg/day and kcal/day in children with CP [6,8].

One of the CP-specific predictive equations was proposed by Krick et al. [8], incorporating factors such as muscle tone, activity level, and growth factor into energy requirement estimation. A recent study performed by Rakhmaeva et al. [34] compared energy intake with energy requirements estimated using the Krick equation in children with spastic CP and found that energy intake was comparable to the estimated energy requirements. Some studies have developed CP-specific models incorporating body weight, TSF, or TBW [4,7,9,12]. However, these models have not demonstrated sufficient precision or external validity for individualized clinical use [4,7,8,9].

With respect to TEE, studies using DLW reported lower values in children with CP compared with TDC [13,16,18] reinforcing the role of reduced physical activity and functional limitations as major determinants of TEE. The study carried out by Rieken et al. [9] analyzed TEE in children classified as GMFCS IV vs. V and demonstrated that TEE is higher in the GMFCS IV group. However, this result should be interpreted carefully since the sample size was very small in the GMFCS IV group (n = 6). Sullivan et al. [2] analyzed TEE in a group of gastrostomy-fed compared with orally fed children with CP. The orally fed group showed higher TEE than that of the gastrostomy-fed group. Similar results were demonstrated in other studies that analyzed REE in both groups [9].

Although BIA proved useful for nutritional assessment, it was considered a complementary tool for estimating EE because the accuracy of BIA was influenced by factors such as spasticity, contractures, postural deformities, and hydration status [9,12,15,17,22,26]. On the other hand, BIA, widely used for assessing body composition and estimating FFM, also showed limitations when applied as the primary method for estimating REE, particularly in the presence of spasticity, contractures, postural deformities, and hydration alterations.

Finally, methods based on height or weight (kcal/cm/d or kcal/kg/d) and hormonal models incorporating ghrelin offer promising complementary strategies. Height-based approaches proposed by Culley et al. [23] suggested energy requirements of 14.7 kcal/cm/day in children without motor dysfunction, 13.9 kcal/cm/day in ambulatory children with motor dysfunction, and 11.1 kcal/cm/day in non-ambulatory patients. These approaches may provide practical alternatives in clinical settings where IC or DLW are not feasible; however, these empirical methods require further validation before routine clinical use [5,10,15,22,28].

Although IC and DLW remain reference techniques, their limited availability in routine clinical practice partly explains the continued reliance on predictive equations, despite their variable accuracy and limited individual-level applicability. Based on this evidence, this scoping review provides a practical clinical algorithm for estimating REE and TEE in children with CP. Direct validation of BIA-derived REE estimates against IC remains scarce, and no sufficiently large external validation studies have examined performance across GMFCS levels and feeding modalities. These findings underscore the need for clinically feasible, validated approaches that integrate motor function, body composition, and nutritional status when estimating energy requirements in this population.

This review and the available evidence have several limitations. Considerable heterogeneity was observed regarding sample size, age, CP subtype, GMFCS distribution, feeding method, and methods used to estimate EE, limiting direct comparisons across studies. Furthermore, most studies included relatively small sample sizes. The available evidence is also largely derived from highly selected populations, predominantly children with severe motor impairment (GMFCS IV–V), whereas ambulatory children and those with milder and lower functional limitations (GMFCS I–III) remain underrepresented. Furthermore, no formal risk-of-bias assessment was conducted, and the heterogeneity of populations, outcomes, and assessment methods precluded quantitative synthesis. Finally, the included studies in this review assessed different outcomes, including REE, TEE, and BMR, which may have contributed to variability in the interpretation of results.

## 5. Conclusions

Energy expenditure in children and adolescents with CP varies according to age, body size, body composition, nutritional status, feeding method, and GMFCS levels. IC remains the reference method for assessment of REE, whereas DLW is the reference method for measuring TEE, which is preferred when available for individualized assessment.

When reference methods are unavailable, energy estimates should be interpreted cautiously and adjusted through longitudinal monitoring of growth, body composition, and clinical outcomes. Predictive equations developed for TDC should be avoided in children with CP because they tend to overestimate energy estimates, whereas CP-specific equations and models for this population provide estimates that are closer to IC measurement.

The evidence analyzed in this scoping review highlights several important methodological limitations and knowledge gaps regarding the assessment of EE in children with CP. In particular, the validation of BIA against IC for estimating REE remains an understudied area; further studies are needed to validate CP-specific equations against reference methods. Moreover, future studies should better account for the heterogeneity of this population when assessing EE, particularly regarding gross motor function, physical activity, body composition, nutritional status, feeding method, spasticity, and hydration status, using longitudinal designs with larger sample sizes.

## Figures and Tables

**Table 1:** Characteristics of the 19 studies included in the scoping review.

Authors Country	Outcome	GMFCS	Age (Years)	n	Method Used	Children with CP				TDC		
						REE (kcal/Day)	REE (kcal/kg/Day)	TEE (kcal/Day)	Energy Intake (kcal/Day)	REE (kcal/Day)	TEE (kcal/Day)	Main Findings
Arrowsmith et al. [3] Australia	REE	V	3 to 18	56	IC	786.3	-	-	1319	1327	-	1. Predictive equations are unreliable. 2. IC is recommended for individualized management.
Azcue et al. [12] Canada	REE	V	2 to 16	13	IC	760	-	-	855	1141	-	1. REE in children with SQCP is not reliably predicted by standard equations. 2. Measurement of REE by IC is recommended for accurate nutritional management in severe SQCP children.
Bell et al. [13] Australia	REE	I–II	5 to 12	16	IC	1075	-	-	-	1679	-	1. Standard equations may overestimate energy needs. 2. Individualized energy requirement estimation is recommended.
	TEE				DLW	-	-	1676	-	-	1985	
Borsani et al. [4] Italy	REE	V	1 to 19	100	IC	806.5	41.96	-	1146	-	-	1. Predictive equations for TDC perform poorly in CP. 2. IC remains the gold standard.
García Contreras et al. [15] Mexico	REE	V	6 to 13	13	IC	775	54.6	-	-	1090	-	1. BIA may provide more accurate and stable REE estimations. 2. Equations for TDC have limited applicability in CP. 3. IC or BIA recommended for accurate estimation.
	REE				BIA	849	58.1	-	-	1182	-	
	TEE				BIA	-	-	1189	-	-	1880	
García Iñiguez et al. [22] Mexico	REE and TEE	I–V	2 to 16	79	BIA	894	54.9	1236	-	-	-	1. EE based on height is a practical and reliable method for estimating energy requirements.
Krick et al. [8] USA	TEE	-	<1 to 18	30	Krick equation	-	79.4	-	-	-	-	1. Krick method is more useful to predict energy needs for children with severe CP than the traditional RDA method.
					RDA equation	-	90.6	-	-	-	-	
Lee et al. [6] China	REE	-	3 to 18	15	IC	800.5	-	-	935.3	-	-	1. Predictive equations substantially overestimate EE in spastic CP. 2. IC provides a more accurate assessment.
					Culley et al. [23]	1213.4	-	-	-	-	-	
					Krick et al. [8]	1603.1	-	-	-	-	-	
					RDA	1928.1	-	-	-	-	-	
Penagini et al. [7] Italy	REE	-	6 to 18	54	IC	876	40.0	-	-	-	-	1. Common equations are inaccurate in SQCP. 2. IC remains a reference standard.
Rieken et al. [9] Netherlands	TEE	IV–V	2 to 19	61	DLW	-	-	GMFCS IV: 1619 kcal/day GMFCS V: 1103 kcal/day	-	-	-	1. New TBW-based models substantially improve TEE estimation accuracy.
Rodríguez-Hernández et al. [24] Mexico	REE	IV	4 to 8	19	IC	Yogurt group: 882 Kefir group: 992.72	-	-	-	-	-	1. No significant differences between groups in REE.
Sert et al. [25] Turkey	BMR	-	5 to 12	41	BIA	413.44	-	-	-	555.02	-	1. BMR was higher in the control group.
Stallings et al. [16] USA	REE	-	2 to 18	61	IC	827	-	-	1332	963	-	1. Energy prescriptions should be individualized based on measured REE rather than generalized population formulas.
	TEE			32	DLW	-	-	963	1394	-	1657	
Sullivan et al. [2] England	REE	I–V	1 to 18	40	IC	693.8	GT: 35.4 kcal Oral: 41.8 kcal	-	-	-	-	1. Children with severe spastic CP show lower REE/TEE. 2. GT feeding increases the risk of excess fat accumulation if not adjusted to measured EE. 3. Energy prescriptions should be individualized.
	TEE				DLW	-	GT: 43.7 kcal Oral: 62.8 kcal	856.5	-	-	-	
Sung et al. [26] Korea	BMR	I–V	6–17	100	BIA	929.7	-	-	-	1112.7	-	1. BMR progressively decreased from GMFCS I to V.
Tomoum et al. [17] Egypt	BMR	I–V	2 to 8	40	BIA	688.7	-	-	-	735.1	-	1. Altered body composition reflects compromised nutritional and metabolic status.
Walker et al. [18] Australia	TEE	I–V	2.9 to 4.4	32	DLW	-	-	1242.8	-	-	1532.2	1. Existing equations for healthy children are inaccurate, especially in non-ambulant children.
Walker et al. [19] Australia	TEE	I–V	2.9 to 4.4	31	DLW	-	-	1228.9	1106	-	1528.6	1. Energy intake was less than TEE in children with mild CP and TDC.
Zenitani et al. [27] Japan	REE	-	4.8 to 11.1	20	IC	574.5	34.9	-	607	-	-	1. Total ghrelin was a strong predictor of REE in CP and SMID. 2. Ghrelin measurement may serve as a practical nutritional biomarker for predicting EE.

Abbreviations: BIA, bioelectrical impedance analysis; BMR: basal metabolic rate; CP, cerebral palsy; DLW, doubly labeled water; EE, energy expenditure; GMFCS, Gross Motor Function Classification System; GT, gastrostomy tube; IC, indirect calorimetry; RDA, Recommended Dietary Allowance; REE, resting energy expenditure; SMID, severe motor and intellectual disability; TEE, total energy expenditure.

## Data Availability

No new data were created or analyzed in this study. Data sharing is not applicable to this article.

## Supplementary Materials

The following supporting information can be downloaded at: https://www.mdpi.com/article/10.3390/healthcare14193295/s1, Table S1: Equations for the search strategy according to databases; Table S2: Methodological characteristics of the included studies.

## Abbreviations

The following abbreviations are used in this manuscript:

BIA	Bioelectrical impedance analysis
BMR	Basal metabolic rate
CP	Cerebral palsy
DLW	Doubly labeled water
EE	Energy expenditure
FFM	Fat-free mass
GMFCS	Gross Motor Function Classification System
IC	Indirect calorimetry
ICC	Intraclass correlation coefficient
RDA	Recommended dietary allowance
REE	Resting energy expenditure
SQCP	Spastic quadriplegic cerebral palsy
TBW	Total body water
TDC	Typically developing children
TEE	Total energy expenditure
TSF	Triceps skinfold thickness
WHO	World Health Organization
