# Supplementary material for: Assessment of Methods to Estimate Resting and Total Energy Expenditure in Children and Adolescents with Cerebral Palsy: A Scoping Review
Source: Healthcare (Basel). 2026 Oct 2;14(19):3295. doi: 10.3390/healthcare14193295 (PMC13665139; doi:10.3390/healthcare14193295)
Supplement: Supplementary file 1 [file healthcare-14-03295-s001.zip › healthcare-4514038-supplementary.pdf]

Supplementary Material

Table S1. Equations for the search strategy according to databases

Database	Equation	Number of studies
Pubmed	(("cerebral palsy"[All Fields] OR "cerebral palsy"[Title/Abstract]) AND ("basal metabolism"[All Fields] OR "basal metabolism"[Title/Abstract] OR "energy metabolism"[Title/Abstract] OR "energy metabolism"[All Fields] OR "basal metabolic rate"[All Fields] OR "basal metabolic rate"[Title/Abstract] OR "resting metabolic rate"[Title/Abstract] OR "resting metabolic rate"[All Fields] OR "basal energy expenditure"[All Fields] OR "basal energy expenditure"[Title/Abstract] OR "resting energy expenditure"[Title/Abstract] OR "resting energy expenditure"[All Fields] OR "total energy expenditure"[All Fields] OR "total energy expenditure"[Title/Abstract] OR "energy expenditure"[Title/Abstract] OR "energy expenditure"[All Fields]) AND ("methods to measure"[All Fields] OR "methods to measure"[Title/Abstract] OR "predictive equations"[Title/Abstract] OR "predictive equations"[All Fields] OR "equations"[All Fields] OR "equations"[Title/Abstract] OR "indirect calorimetry"[Title/Abstract] OR "indirect calorimetry"[All Fields] OR "bioelectrical impedance"[All Fields] OR "bioelectrical impedance"[Title/Abstract] OR "methods"[Title/Abstract] OR "methods"[All Fields] OR "doubly labeled water"[All Fields] OR "doubly labeled water"[Title/Abstract] OR "calorimetry"[All Fields] OR "calorimetry"[Title/Abstract]) AND ("child"[All Fields] OR "child"[Title/Abstract] OR "children"[Title/Abstract] OR "children"[All Fields] OR "adolescents"[All Fields] OR "adolescents"[Title/Abstract])) AND ((humans[Filter]) AND (english[Filter] OR spanish[Filter]))	121
Scopus	TITLE-ABS-KEY("cerebral palsy") AND TITLE-ABS-KEY("basal metabolism" OR "energy metabolism" OR "basal metabolic rate" OR "resting metabolic rate" OR "basal energy expenditure" OR "resting energy expenditure" OR "total energy expenditure" OR "energy expenditure") AND TITLE-ABS-KEY("methods to measure" OR "predictive equations" OR "equations" OR "indirect calorimetry" OR "bioelectrical impedance" OR "methods" OR "doubly labeled water" OR "calorimetry") AND TITLE-ABS-KEY("child" OR "children" OR "adolescents") AND LANGUAGE("English" OR "Spanish")	154
SciELO	("cerebral palsy") AND ("basal metabolism" OR "energy metabolism" OR "basal metabolic rate" OR "resting metabolic rate" OR "basal energy expenditure" OR "resting energy expenditure" OR "total energy expenditure" OR "energy expenditure") AND ("methods to measure" OR "predictive equations" OR "equations" OR "indirect calorimetry" OR	2

	"bioelectrical impedance" OR "methods" OR "doubly labeled water" OR "calorimetry") AND ("child" OR "children" OR "adolescents")	
EBSCO	"cerebral palsy" AND "basal metabolism" OR "energy metabolism" OR "basal metabolic rate" OR "resting metabolic rate" OR "basal energy expenditure" OR "resting energy expenditure" OR "total energy expenditure" OR "energy expenditure" AND<br>methods to measure" OR "predictive equations" OR "equations" OR "indirect calorimetry" OR "bioelectrical impedance" OR "methods" OR "doubly labeled water" OR "calorimetry" AND "child" OR "children" OR "adolescents"	20
Embase	(('cerebral palsy':ti,ab,kw AND 'basal metabolism':ti,ab,kw OR 'energy metabolism':ti,ab,kw OR 'basal metabolic rate':ti,ab,kw OR 'resting metabolic rate':ti,ab,kw OR 'basal energy expenditure':ti,ab,kw OR 'resting energy expenditure':ti,ab,kw OR 'total energy expenditure':ti,ab,kw OR 'energy expenditure':ti,ab,kw) AND 'predictive equation':ti,ab,kw OR 'equation':ti,ab,kw OR 'indirect calorimetry':ti,ab,kw OR 'bioelectrical impedance':ti,ab,kw OR 'doubly labeled water':ti,ab,kw OR 'calorimetry':ti,ab,kw) AND 'child':ti,ab,kw OR 'adolescent':ti,ab,kw<br>Filters: language (Spanish and English), study design (cross-sectional, longitudinal, case-control), age (0-19 years)	77
**TOTAL**		**374**

Table S2. Methodological characteristics of the included studies

Author	EE method/device	Comparator / reference	Statistical approach relevant to EE	Equation/Model Predictive	Main methodological finding / limitation
Arrowsmith et al., 2012 [3]	IC: Datex Deltatrac II MBM-200 Metabolic Monitor (Datex-Engstrom Division Instrumentarium Corp., Helsinki, Finland)	CP vs healthy comparison group: REE in relation to predicted values; longitudinal change after nutritional rehabilitation	Linear regression; group comparisons; longitudinal comparisons	REE equations reported in study	REE was lower and more variable in severe CP and could not be accurately predicted at the individual level. Follow-up sample was small (n=14).
Azcue et al., 1996 [12]	IC: 2900 Metabolic Cart Sensormedics; BIA: BIA 101 (RJL Systems Inc., Detroit. Mich.)	SQCP vs controls; measured REE vs predicted REE; BIA-derived body composition evaluated against isotope-dilution measures	Student's *t*-test; linear regression; ANCOVA; correlations	WHO prediction of REE; FFM derived from TBW	REE was significantly lower in SQCP and inadequately predicted by standard equations. Small, selected sample of nonambulatory SQCP limits generalizability.
Bell et al., 2010 [13]	IC: Deltatrac II (Datex, Helsinki, Finland); DLW for TEE; Cosmed K4b² (Cosmed, Srl, Italy)	Ambulatory CP vs TDC; REE and TEE between groups	Group comparisons; Pearson correlations; regression	—	REE was similar between groups, whereas TEE was lower in CP. This is primarily a physiological group comparison rather than a validation of IC or DLW.
Borsani et al., 2023 [4]	IC: Open-circuit calorimeter (Vmax 29, Sensor Medics, Yorba Linda, CA)	REE measured by IC vs commonly used prediction equations; development of CP-specific models	Bland–Altman; proportional-bias analysis; concordance analysis;	Standard predictive equations + newly developed CP-specific models	Standard equations showed limited individual accuracy and proportional bias.

			regression/model development		Population restricted to GMFCS V; newly developed models require external validation.
García-Íñiguez et al., 2018 [22]	BIA: QuadScan 4000 (Bodystat Ltd, Isle of Man, British Isles, England.)	EE across age/sex/anthropometric and body-composition characteristics; no IC/DLW reference	ANOVA with Bonferroni; Pearson correlations	Device-derived REE and TEE; equation not reported	Associations of estimated EE with age, anthropometry and body composition were examined, but BIA-derived EE was not validated against a reference EE method.
García-Contreras et al., 2015 [15]	BIA: QuadScan 4000 (Bodystat Ltd, Isle of Man, British Isles, England); IC: Ree Vue 8100 (model 8100, Salt Lake City, Utah)	BIA-derived EE vs IC; CP vs healthy children; repeated measurements during nutritional recovery	Paired *t*-test; Pearson correlation; ROC analysis	BIA-derived estimates; equation not reported	BIA and IC produced different REE estimates, and variability changed during nutritional recovery. Small sample (n=13), GMFCS V and moderate/severe malnutrition limit generalizability.
Krick et al., 1992 [8]	Predictive calculation; no IC/DLW reference	Krick method vs RDA vs discharge energy prescription	Paired *t*-test; Pearson correlation; multiple regression	Krick equation; RDA method; BMR based on body-surface area	Krick method was closer to the clinically established discharge prescription than RDA. Retrospective design and absence of an objective reference

					measurement of EE limit validation.
Lee et al., 2011 [6]	IC: MedGraphics CardiO_2 (MedGraphics, St. Paul, Minnesota)	Measured EE by IC vs BSA/Culley, RDA and CP-specific/Krick approaches	Friedman test; paired *t*-tests; ICC	Culley/BSA, RDA, Krick/CP-specific calculations; IC calculated using Weir equation	Predictive approaches substantially overestimated EE relative to IC. Small institutionalized sample (n=15), spastic CP and sedation limit generalizability.
Penagini et al., 2018 [7]	IC: Open-circuit calorimeter (Vmax 29, Sensor Medics, Yorba Linda, CA)	Five predictive equations vs REE measured by IC	Paired Student's *t*-test; Bland–Altman; Pearson correlation; regression/model development	WHO, Schofield, Oxford, Harris–Benedict and other standard equation evaluated; new CP-specific formula developed	Standard equations showed clinically relevant individual prediction error. New CP-specific formula was developed but requires external validation. Sample restricted to SQCP/GMFCS V and lacked detailed body-composition assessment.
Rieken et al., 2011 [9]	DLW: isotope-ratio mass spectrometry (Thermo Fisher); BIA: Bio-impedance Analyzer (Akern SRL, Florence, Italy)	Existing CP-specific body-composition/TEE equations vs reference measurements; newly developed models vs measured values	ICC; Bland–Altman; regression; R^2; SEE/RMSE; bootstrap/model validation procedures	Existing CP-specific equations including Krick plus newly developed TBW-based TEE model	Existing equations showed inadequate accuracy; TBW-based models improved prediction. Height-measurement error and restriction to severe neurological impairment/GMFCS

					IV–V limit generalizability.
Rodríguez Hernández et al. 2022 [23]	IC: CardioCoach calorimeter (KORR Medical Technology, Salt Lake City, UT, USA)	Kefir vs yogurt control group; REE assessed at baseline and after 7 weeks	Student's *t*-test for independent and paired samples; correlation analyses	—	REE was directly measured by IC, but the study was designed to assess the effects of kefir rather than to validate the EE method. No significant between- or within-group changes in REE were observed. Small sample (19 completing participants), restricted to GMFCS IV.
Sert et al. 2009 [24]	BIA: BIA 450 Bio Impedance Analyzer (Biodynamics Corp., Seattle, WA, USA)	Children with spastic CP vs typically developing controls; no IC/DLW reference	Independent-samples t-test	BMR estimated by BIA device; equation not reported	BIA-derived BMR was significantly lower in children with CP than in controls. However, BMR was estimated by the device and was not validated against IC or another reference EE method.
Stallings et al., 1996 [16]	IC: Gould 2900 or SensorMedics 2900Z (Sensor Medics, Yorba Linda, CA);	SQCP vs healthy controls; measured REE vs predicted REE; TEE between groups	ANOVA; ANCOVA; multiple regression	WHO-predicted REE	SQCP participants had lower REE/TEE, particularly after accounting for body composition. DLW

	DLW(Aldrich, Milwaukee; EG&G Mound, Miamisburg)				was available only in a subgroup and the study primarily compared populations rather than methods.
Sullivan et al., 2006 [2]	IC: ventilated hood system Deltatrac (Datex-Ohmeda Instrumentarium Corporation, Helsinki, Finland); DLW (Cambridge Isotope Laboratories, Andover, MA, USA)	Gastrostomy-fed vs orally fed children; baseline/follow-up energy balance	Wilcoxon rank-sum; linear regression/adjusted comparisons	—	GT-fed children had lower RMR/TEE despite greater fat stores. IC and DLW were measurement methods rather than methods being validated against one another; sample size was limited.
Sung et al., 2017 [25]	BIA: multi-frequency 50 bioelectrical impedance analyzer (InBody S10; InBody, Co., Ltd., Seoul, Korea)	Children with CP by GMFCS/ambulatory status and typically developing controls	Independent t test; one-way ANOVA; age adjusted ANCOVA; Bonferroni; partial correlations; multiple regression	BMR estimated by BIA device; equation not reported	Nonambulatory children (GMFCS IV–V) had significantly lower BMR and several lean-tissue measures. No IC or DLW reference was used, so BIA-derived BMR was not validated as a measure of EE. Authors themselves state that further study is required before BIA can be considered a valid

					nutritional assessment tool in CP.
Tomoum et al., 2010 [17]	BIA: Holtain Body Composition Analyzer – Holtain's BCA (Holtain ltd., UK, Wales, Crosswell)	CP vs healthy controls; comparisons according to severity	Student's *t* test for parametric data; *z*-test for non-parametric data; Pearson χ^2; Pearson correlation	BMR derived from BIA prediction; equation not reported	BMR was significantly lower in children with CP than controls. However, BMR was an output calculated by BIA, not measured by IC, and there was no comparison with a criterion EE method. Therefore, this study cannot establish validity of BIA-derived BMR for EE.
Walker et al., 2012 [18]	DLW: Dual Inlet Isoprime isotope ratio mass spectrometer (Isoprime Dual Inlet IMRS e IonVantage Software, Isoprime, Manchester, UK)	CP vs TDC; ambulant vs marginally/nonambulant CP; measured energy requirements in relation to functional severity	ANOVA; Tukey post-hoc; regression; methodological comparisons reported in study	Predictive energy-requirement approaches evaluated against DLW-derived requirements	Energy requirements varied substantially with functional ability and were lower in CP, particularly nonambulant CP. Some CP subgroups were small and age range was limited to preschool children.
Walker et al., 2013 [19]	DLW: Dual Inlet Isoprime isotope ratio mass spectrometer (Isoprime Dual Inlet IMRS–IonVantage Software, Isoprime)	Energy intake from modified 3-day weighed food record vs TEE measured by DLW	EI–TEE comparison; EI/TEE ratio; bias expressed as % of TEE; Bland–Altman analysis; group comparisons	—	DLW was not being validated; it served as the criterion/reference method for validating dietary EI. The food record showed acceptable group-level

					agreement, particularly in moderate–severe CP.
Zenitani et al., 2021 [26]	IC: AE300S (Minato Medical Science, Osaka, Japan) in patients without a tracheostomy and CCM Express (Medical Graphics Corporation, St. Paul, MN, USA) in patients with a tracheostomy	Associations between measured REE/REE per BW and plasma ghrelin, anthropometry, nutritional markers, and EI; comparisons among CP, muscular disease, and controls	Correlation analyses and regression analyses	Ghrelin-based predictive relationship/model but this was not developed/validated as a clinical EE prediction equation	A methodological consideration is that two different calorimeters were used according to tracheostomy status, and some patients were lightly sedated before IC. Total ghrelin was strongly associated with REE/BW, but the proposed predictive relationship was not externally validated against an independent sample/model; small and heterogeneous clinical population.

Abbreviations: ANCOVA, analysis of covariance; ANOVA, analysis of variance; BIA, bioelectrical impedance analysis; BMR, basal metabolic rate; BSA, body surface area; BW, body weight; CP, cerebral palsy; DLW, doubly labeled water; EE, energy expenditure; EI, energy intake; FFM, fat-free mass; GMFCS, Gross Motor Function Classification System; GT, gastrostomy tube; IC, indirect calorimetry; ICC, intraclass correlation coefficient; RDA, Recommended Dietary Allowance; REE, resting energy expenditure; RMR, resting metabolic rate; RMSE, root mean square error; ROC, receiver operating characteristic; SEE, standard error of the estimate; SMR, sleeping metabolic rate; SQCP, spastic quadriplegic cerebral palsy; TBW, total body water; TDC, typically developing children; TEE, total energy expenditure; WHO, World Health Organization.
